# President’s Address

**Published:** 1902-06-15

**Authors:** C. R. Rowley


DENTAL REGISTER.

Vol. LVI. June 15, 1902. No. 6.

### PRESIDENT'S ADDRESS.

BY C. R. ROWLEY, D.D.S.

Read before the Southwestern Michigan Dental Society, April 8, 1902.

The intent of this paper is to give a brief sketch of some of the older dentists who have been interested in the affairs of this society.

You may not realize the difficulty of compiling a paper of this nature. Out of forty or fifty letters sent out asking for information and data, only six were answered giving the desired information. Therefore the writer is compelled to use his own knowledge and memory of men whose names he has taken the liberty to mention.

Beginning at Detroit we first think of Dr. George L. Field, who commenced the practice of dentistry very early in life in the rural districts of the State of Missouri, but moved to the beautiful city of Detroit in the year 1857, and has been in continual practice there up to and including the present time. Dr. Field has always taken an active part in local, State and national dental societies. He is a forcible speaker, full of wit and good sense, and always commands the attention of his listeners when speaking. He has reached the half century mark in the practice of dentistry, and has always enjoyed the distinction of being a leader instead of a follower in the rapid advancement of the dental profession.

For many years Dr. Hiram Benedict was an active dentist of Detroit, locating in that city about the year 1850. He enjoyed a large and lucrative practice up to the year 1875, when unfortunate business ventures wiped away his accumulation. Thinking he could best retrieve his lost fortune in a larger city, he moved with his wife and two daughters to Chicago, where in a few years physical ailments befell him, and he was compelled to relinquish his profession and take up the sale of dental supplies, which he continued up to the time of his death, January 15, 1901. He expressed his interest in our society work in many ways.

Dr. C. H. Oakman, a graduate of the Chicago Dental College, is one of our contributors. He is a young man with a large and growing practice, and plans ultimately to specialize in oral surgery. He has a liking for this work and has had special training under Dr. Brophy, of Chicago, and is at present taking special work in the Detroit Medical College for this purpose.

Dr. J. L. Young, another young man of Detroit, has made contributions to our programs. He is an enthusiastic crown and bridge worker and likewise a fine operator, and has a brilliant future before him.

At Ypsilanti lives Dr. Jno. A. Watling, though born in Illinois in 1839. He moved to Michigan early in life and began the study of dentistry in the office of Dr. Carlton, in Ypsilanti, in the fall of 1856. In the spring of 1858 he went to Cincinnati, Ohio, and entered the office of Drs. Knowlton and Taft, where he remained two years, attending lectures of the Ohio Dental College, graduating in the spring of 1860, soon thereafter returning to Ypsilanti, where he has remained to the present time. When the University of Michigan opened the dental department in the year 1875, Dr. Watling was secured as one of the faculty, and he has continued teaching dentistry continuously since that date. He is a frequent visitor to this society. The Watling instruments are standard and will perpetuate his name.

At Ann Arbor we find Prof. J. Taft, dean of the dental department of the University. Dr. Taft is so well known everywhere that I need not go into details about him. He has lived and practiced in Cincinnati until last summer when he moved to Ann Arbor. Prof. Taft has often visited this society. He is one of the *stalwarts* in dentistry, a writer, teacher, editor and operator of unquestioned ability for more than forty years.

Prof. Nelville S. Hoff is also a member of the dental faculty at Ann Arbor. He is a comparatively young man and we look upon him as a loyal supporter of this organization. As editor of the DENTAL REGISTER he publishes the proceedings of our meetings.

Drs. W. H. Dorance and W. H. Jackson, of Ann Arbor, are among the older members of the dental profession, but they have not attended the meetings of this society.

As we reach the city of Jackson, instantly the name ot Robinson comes to our mind, but we regret that we have been unable to obtain dates and authentic matter concerning the life of Dr. Jerry Robinson. We must therefore speak from memory and personal knowledge. Dr. Robinson was known to the profession as "Uncle

Jerry, the poet dentist." If you have filed away your dental journals and can turn the pages back ten, fifteen, twenty and twenty-five years, you will find many poems, all gems of thought, written by "Uncle Jerry." It would be difficult to find in western Michigan a man more honored and respected. Dr. Robinson at the time of his death was honored for his professional ability, respected for his moral worth and loved for the inspiration of good cheer he imparted to others.

Jackson was formerly the home of Dr. C. S. Case, the well-known teacher of orthodontia in the Chicago College of Dental Surgery. Dr. Case is an honorary member of this society, and has favored us with papers, illustrations of cases that have come under his care for treatment.

Jackson is also the home of Dr. C. Blair Blackmarr, a graduate of Ann Arbor. He has practiced in his present location about fifteen years. Dr. Blackmarr frequently responds to the call of the secretary when requested to furnish papers for this society.

Dr. Geo. H. Mosher has been for thirty-five years a practitioner in Jackson. He has never visited this society, but I am told that he has not missed a meeting of the State society in twenty-five years, and has been the custodian of the funds for nearly that length of time. On account of his wife's health he is now a resident of New Mexico.

Albion is the home of Dr. Wright, not very well known by our members, but highly esteemed by the citizens of Albion.

Albion is also the home of Dr. C. H. Warboys, an esteemed member of our society, a young man with ideas and a conviction that he must defend them ; full of life and always ready to lead the singing.

At Marshall, is located Dr. Eggleston, a man of considerable genius, but he allows his light to shine under a bushel, as he is never seen in any gathering of dentists. The dental atmosphere of Marshall is musty, and we are glad to get away and on to the city of Battle Creek.

Our entire paper could profitably be written of the dentists in this hustling city. D. C. Hauxhurst, now deceased, was to Battle Creek what Atkinson was to New York in years gone by. Dr. Hauxhurst first located in Galesburg in 1870, when it was the writer's privilege to be one of his students. About the year 1875 he moved to Battle Creek. He married one of Battle Creek's fair daughters in 1882, and while on his wedding journey died in Paris, France. With his death the press lost a valuable contributor, the profession a respected and honored member, and Battle Creek a distinguished citizen.

Dr. A. T. Metcalf, formerly of Kalamazoo, but for the past fifteen years a citizen of Battle Creek, was the "power behind the throne" in securing the passage of the Michigan dental law. He is not now in practice, but attends our meetings when he can.

Dr. R. M. Speer, the Vice President of this society, has adopted the motto "Never miss a meeting," and we find him one of the principal factors in giving this society its present enviable reputation.

Dr. S. M. Fowler, ex-president of the Michigan State Dental Society, is an active member of this body and one of the most successful toast masters in the State of Michigan. Like Detroit, Battle Creek has too many dentists to mention in one paper.

In the city of Kalamazoo we have several men reaching out toward fifty year's active practice. Drs. Holmes,

Westbrook and Bannister. The writer tried to get dates of their past history, but was not successful. Dr. E. A. Honey, for several years our efficient secretary, has a large and remunerative practice here. Drs. Siddall, Cook, Wise, Sizeln and others are located here. They are all able men and have furnished this society with excellent papers and participated in its proceedings.

At Lawton we look in upon our genial secretary, Dr. C. W. Johnson. To all outward appearances the climate, fruit and dental practice agree with him.

At Paw Paw, county seat of Van Buren County, we find Dr. W. C. Y. Ferguson, a strong believer in dental societies and ardent supporter of the Southwestern Michigan.

Dr. N. C. Hooker, now of San Jose, Cal., located in Paw Paw, about the year 1870, where he remained several years before going to his present location. Drs. Robinson and Ward, the former the son of "Uncle Jerry" of Jackson, were located in Paw Paw about the year 1880 to '85. Dr. Robinson moved to Grand Rapids, where he died about two years ago. Dr. Ward moved to Kalamazoo, where consumption carried him away soon after his arrival in that city. Dr. O. E. Lanphear is now practicing here.

At Decatur, where a pleasant meeting of this society was held last year, is the home of Dr. N. E. Hooper, ex-president of this society. Dr. Hooper is about forty years of age. Laying the foundation of his professional knowledge as a student of Dr. Swazy, of Chicago, he has by close application continued to keep abreast the times. Dr. Hooper's success as an operator has cleared the field from competitors. Speaking of competitors reminds us of the time when Decatur tried to support five dentists, Drs. C. S. and C. R. Rowley, Drs. Chas. and Howard Crandall and a Dr. King. Dr. C. S. Rowley located in Decatur in the year 1859, under the firm name of Drs. Rowley and Day, Dr. W. W. Day hailing from Lawton, and Dr. Rowley from Cleveland, Ohio. Dr. Day retired from the firm in 1867, and Dr. C. R. Rowley succeeded him, when the firm name was changed to Dr. Rowley & Son. Dr. C. R. Rowley continued to practice in Decatur until December 1, 1873, when he moved to Niles, Mich. The Drs. Crandall moved to Northern Michigan and Dr. King to London, England.

At Dowagiac is the home of Drs. T. G. Rix, F. H. Essig and F. H. Codding. Dr. Rix located in Dowagiac about the middle of the last century. For a number of years there was a firm name known as the Rix Bros. This, however, was discontinued about the year 1870, the brother withdrawing and moving to another State, Dr. T. G. Rix continuing the business up to the present time. During the years of 1889 and '90 Dr. Rix had entire charge of the clinical department of the American (now Northwestern University) College of Dental Surgery. He is one of the originators of this society, and claims to be the first dentist in Michigan to make crown and bridge-work, a claim the writer has never heard disputed.

Dr. F. H. Essig, ex-president of the Southwestern Michigan and present secretary of the State society, located in Dowagiac about fifteen years ago, and is constantly working at and holding on to everything good pertaining to dentistry. Dr. F. H. Codding, now of piscatorial fame, is the junior dentist of the place.

The Michigan Central Railroad was built into Niles in the year 1850, and with it came Dr. George A. Howe, a dentist who gave the most of his attention to mechanical dentistry.

In the year 1871 Dr. Howe made a gold crown (central incisor) in the presence of the writer. In the year 1888 Dr. Howe concluded to get the degree of doctor of dental surgery. He matriculated at the American College of Dental Surgery. The strain, however, was too great for his advanced years, and he died the winter of 1899 of brain fever. Dr. J. B. Glenn is one of the oldest dental practitioners in Western Michigan. He is holding his own at the age of seventy-two. Dr. Perry Hanson graduated at Ann Arbor the spring of 1886, located in Niles, October 1st the same year, and still continues in that city. Dr. Hanson has built for himself a beautiful home, and is the first dentist in Southwestern Michigan to ride in his own automobile.

Benton Harbor is the home of Dr. Sumter M. White, reasonably credited with the paternal parentage of this organization. About twenty-five years ago Dr. White commenced the practice of dentistry at White Pigeon, this State, a few years later moving to Bangor, where for several years it was a great struggle to keep the wolf from the door. He located in Benton Harbor, November 6, 1876, where he has now one of the best equipped offices in the State and lives on the sunny side of "Easy Street." Dr. H. C. Rockwell, a graduate of the Philadelphia Dental College, located in Benton Harbor in 1875, continued in practice to the year 1898, when he withdrew from the profession and entered commercial life. Dr. S. B. Ellsworth is one of the old veterans in dentistry located at Benton Harbor, but the writer has no data to base a sketch upon.

Over the river on the bluffs of Lake Michigan, two miles distant from Benton Harbor we find St. Joseph, the home of Dr. W. W. Ray, now deceased, long and favorably known to the dentists of Michigan. This is also the home of Dr. E. I. Backus, who had the honor of presiding over this body in the year 1897. Like the Roman of old, the art of speech is not one of his qualifications, but his clinics are valuable and instructive. Dr. S. W. Honey, comparatively a new member, but worthy of more extended notice, is a resident also of this place. Dr. Ray, Jr., has been present at every meeting of this society, a record to be proud of.

Dr. A. C. Runyan at an early day was associated with S. M. White, the two conducting not less than seven offices in the small towns located between South Haven and Benton Harbor. Tiring of this nomadic life, Dr. Runyan in the year 1884 settled in South Haven. The doctor enjoys the distinction of being an ex-president of this society, and seems to be one of those men that time makes but little impression upon, as he has changed but little in the twenty years it has been our privilege to know him. Dr. J. C. Arnold, his associate, is one of the rising young men of our profession and is devoutly attached to this society. At Buchanan, and we close our sketch, is the home of Clark B. Roe, who has many times gladdened our hearts with his songs and good cheer. As head of the executive committee, largely due to his individual efforts, this meeting will be one grand success.

